# Revascularization and cardioprotective drug treatment in myocardial infarction patients: how do they impact on patients' survival when delivered as usual care

**DOI:** 10.1186/1471-2261-6-21

**Published:** 2006-05-04

**Authors:** Alain Vanasse, Josiane Courteau, Théophile Niyonsenga

**Affiliations:** 1 Family Medicine Department, Faculty of Medicine, Université de Sherbrooke, 3001, 12th Avenue North, Sherbrooke (QC), J1H 5N4, Canada; 2 PRIMUS Group, Clinical Research Center, Sherbrooke University Hospital, Sherbrooke (QC), Canada; 3 Stempel School of Public Health, Epidemiology & Biostatistics, Florida International University, Florida, USA

## Abstract

**Background:**

Randomized clinical trials showed the benefit of pharmacological and revascularization treatments in secondary prevention of myocardial infarction (MI), in selected population with highly controlled interventions. The objective of this study is to measure these treatments' impact on the cardiovascular (CV) mortality rate among patients receiving usual care in the province of Quebec.

**Methods:**

The study population consisted of a "naturalistic" cohort of all patients ≥ 65 years old living in the Quebec province, who survived a MI (ICD-9: 410) in 1998. The studied dependant variable was time to death from a CV disease. Independent variables were revascularization procedure and cardioprotective drugs. Death from a non CV disease was also studied for comparison. Revascularization procedure was defined as percutaneous transluminal coronary angioplasty (PTCA) or coronary artery bypass graft (CABG). The exposure to cardioprotective drugs was defined as the number of cardioprotective drug classes (Acetylsalicylic Acid (ASA), Beta-Blockers, Angiotensin-Converting Enzyme (ACE) Inhibitors, Statins) claimed within the index period (first 30 days after the index hospitalization). Age, gender and a comorbidity index were used as covariates. Kaplan-Meier survival curves, Cox proportional hazard models, logistic regressions and regression trees were used.

**Results:**

The study population totaled 5596 patients (3206 men; 2390 women). We observed 1128 deaths (20%) within two years following index hospitalization, of them 603 from CV disease. The CV survival rate at two years is much greater for patients with revascularization, regardless of pharmacological treatments. For patients without revascularization, the CV survival rate increases with the number of cardioprotective drug classes claimed. Finally, Cox proportional hazard models, regression tree and logistic regression analyses all revealed that the absence of revascularization and, to a lower extent, absence of cardioprotective drugs were major predictors for CV death, even after adjusting for age, gender and comorbidity.

**Conclusion:**

Considering usual care management of MI in the province of Quebec in 1998, CV survival is positively correlated to the presence of a revascularization procedure and to the intensity of cardioprotective pharmacological treatment. These results are coherent with data from randomized control trials.

## Background

Cardiovascular events represent a major health burden for Canada and other modern societies and myocardial infarction (MI) accounts for a large percentage of them. MI is a very lethal disease with near 30% of deaths, among which near half occurs before arriving to hospital [1]. The prognosis of this clinical event depends on the patient's acknowledgement of his clinical symptoms and the decision to seek for medical care; on the delay between the first symptoms and the arrival to hospital (onset-to-door); on the emergency care team rapidity of response, and on the swiftness and suitability of the treatment received during hospitalization [2,3], but also after discharge. MI secondary prevention includes all clinical measures taken after the event's occurrence to reduce mortality and/or morbidity of the disease. Cardiovascular secondary prevention includes appropriate revascularization procedures and long term use of known cardioprotective drugs – Acetylsalicylic Acid (ASA), Beta-Blockers, Angiotensin-Converting Enzyme (ACE) Inhibitors, Statins – as well as risk factors reduction with long term lifestyle and/or drug management. Practice guidelines regarding MI management have been widely published in the last decade [4-7].

These clinical guidelines are based on evidence that early revascularization reduces mortality and morbidity [8-12]. However, some clinical trials showed only marginal benefits of revascularization [13,14]. Each of the four pharmacological classes included in the guidelines has also individually demonstrated great benefits to prevent mortality and morbidity in secondary prevention [15-36]. Some combinations of these drug classes have also showed reduced mortality [37-39]. In 2004, Mukherjee et al [40] have demonstrated an improvement in 6-month mortality after an acute coronary syndrome according to a composite appropriateness score defined by a combination of these four drug classes. To our knowledge, little is known about the impact of the combination of these four classes of drugs in addition to revascularization procedure in the general population.

The main objective of this study is to measure in the context of usual care, the impact of surgical (PTCA and CABG) and pharmacological treatments (revascularization and/or the number of type of cardioprotective drugs claimed) on the cardiovascular (CV) survival rate of patients with MI in 1998 in Quebec.

## Methods

### Design

We conducted a population-based cohort study using secondary data analysis from the Quebec's hospital discharge register (MED-ECHO). This register provides administrative data on patients hospitalized in the province of Quebec. Studies confirming the validity of the administrative hospital discharge data concerning MI have previously been published [41,42].

### Studied population

The study population included all patients 65 years and older living in the province of Quebec, who have been hospitalized in Quebec for a MI between January 1^st ^and December 31^st ^1998. We will refer as the "index period" the period defined by the first hospitalization in addition to a 30-day period after discharge from the index hospitalization. We included patients who were hospitalized for acute myocardial infarction (code 410 of the International Disease Classification, 9^th ^revision (IDC-9)) as the main diagnosis. Patients with a MI in the year preceding the index hospitalization were excluded in order to include only new or stable MI cases. Patients from northern and low populated regions (*Northern Quebec*, *Nunavik *and *James Bay Cree Lands) *were excluded as well because of major cultural, social and health care services differences. In order to measure the pharmacological treatments claimed after the MI event, only patients who survived the index period were included in the analyses. A 2-year follow-up period was retained to collect the date and cause of death.

### Data sources

All patients' data were obtained from the Quebec's hospital discharge register (MED-ECHO) and the provincial demographic database. For claimed drugs, we used the provincial pharmacological register administered by the *Régie de l'assurance maladie du Québec *(RAMQ), which covers almost all people aged 65 years or more as well as welfare recipients and people not covered by private drug insurance.

### Studied variables

The main outcome of interest was time to death from CV cause (ICD-9: 410–414, 426–429). Exposure to revascularization was defined as 1 if there was mention of a PTCA or a CABG, as coded in the Quebec's hospital discharge register (Canadian Classification of diagnostic, therapeutic and surgical procedures (CCP) beginning with 480 to 483), for the index period and 0 otherwise. Exposure to medication was defined as the number of cardioprotective drug classes that was claimed within the index period. The following drug classes were considered: *ASA*, *β-blockers*, *ACE inhibitors*, and *Statins*.

Covariates included gender, age, and a comorbidity index. This index is an adaptation of the *D'Hoore index*, which is itself an adaptation of the *Charlson comorbidity index *[43]. The D'Hoore index is a weighted score of comorbid conditions, these conditions being defined by the secondary diagnoses available in the MED-ECHO database in the year preceding the index hospitalization. We adapted this index simply by substracting the quantity 1 to the D'Hoore index, corresponding to the weight associated to the MI comorbid condition.

### Statistical analyses

The chi-square test was used for comparison between proportions, whereas for comparison between means, the t-test or ANOVA was used depending on the number of groups. Survival analyses were done using Kaplan-Meier estimates for unadjusted survival curves (the homogeneity between the curves is tested using the Wilcoxon statistics) and Cox proportional hazard model to obtain death rates adjusted for all the covariates. Logistic regression [44] and regression tree [45,46] analyses were also performed to predict the CV death rate after two years. In regression tree methodology, information on a data set is summarized by dividing the population into a number of subgroups, as homogeneous as possible but distinct with respect to the parameters to predict. The subgroups are identified by a tree-structured figure of binary questions on the predictors. The resulting classification is the most informative one with respect to the parameter in question. Tree-growing techniques [47-49] are particularly suited to handle a large number of variables including some with missing values and to investigate the interactions between them. For all approaches, the potential patient-level predictors were age, gender, the comorbidity index, the presence/absence of a revascularization and the number of cardioprotective drugs claimed within the index period. The trees were pruned at a significance level of 0.001. For all other tests, the significance level used was 0.05. Statistical analyses were done using SAS Release 9.1 and the RTREE program [45,46].

### Ethical considerations

This project was approved by the Sherbrooke University Hospital Ethics Board and the *Commission d'accès à l'information du Québec*.

## Results

A total of 7332 patients 65 years or older have been hospitalized for a MI in Quebec between January 1^st^, 1998 and December 31^st^, 1998. Of those, 172 patients were excluded because they had a MI in the prior year, and 11 because they lived in the Quebec Northern regions. From the remaining 7149 patients, 1553 (21,7%) died at the index period and were removed from the analyses. Therefore, the study population includes 5596 patients, men accounting for 57% (*n *= 3206) of it. Table 1 presents the cohort's characteristics in regard to gender, age and comorbidity index, according to whether they survived the index period or not. Men, younger patients and patients with a lower comorbidity index are more represented in the cohort who survived the index period. Table 2 presents the patients' characteristics according to the revascularization procedure and to the number of cardioprotective drug classes claimed during the index period. This table reveals that men and younger patients, as well as patients with a lower comorbidity index have more chances of receiving a revascularization. On the other hand, age and comorbidity index, but not gender, are statistically associated with the number of cardioprotective drug classes claimed at the index period.

**Table 1:** Description of the population with MI in Quebec in 1998

	*Total population*	*Died at index period*	*Survived index period*	*p-value*
TOTAL	7149	1553 (21.7%)	5596 (78.3%)	

*Gender*				
Men	3982 (55.7%)	776 (50.0%)	3206 (57.3%)	< .0001
Women	3167 (44.3%)	777 (50.0%)	2390 (42.7%)	

*Age*				
Mean ± SD	76.1 ± 7.3	78.9 ± 7.5	75.3 ± 7.0	< .0001
65–74 years	3298 (46.1%)	487 (31.4%)	2811 (50.2%)	< .0001
75–84 years	2823 (39.5%)	674 (43.4%)	2149 (38.4%)	
≥ 85 years	1028 (14.4%)	392 (25.2%)	636 (11.4%)	

*Comorbidity index*				
Mean ± SD (median)	1.8 ± 1.9 (1)	2.4 ± 2.1 (2)	1.6 ± 1.8 (1)	< .0001
0	2345 (32.8%)	333 (21.4%)	2012 (36.0%)	< .0001
1–2	2624 (36.7%)	547 (35.2%)	2077 (37.1%)	
3–4	1538 (21.5%)	427 (27.5%)	1111 (19.9%)	
5–6	499 (7.0%)	181 (11.7%)	318 (5.7%)	
≥ 7	143 (2.0%)	65 (4.2%)	78 (1.4%)	

**Table 2:** Characteristics of MI patients according to exposure to revascularization and medication (n = 5596)

	*Total*	*Men*	*Women*	*Age (years) Mean (± SD)*	*Comorbidity index Mean (± SD)*
*Revascularization*					
No	4473	2505 (56.0%)^a^	1968 (44.0%)	76.2 (± 7.2)^b^	1.7 (± 1.8)^b^
Yes	1123	701 (62.4%)	422 (37.6%)	72.0 (± 5.2)	1.3 (± 1.5)
PTCA	767	470 (61.3%)	297 (38.7%)	72.1 (± 5.4)	1.1 (± 1.4)
CABG	356	231 (64.9%)	125 (35.1%)	71.6 (± 4.7)	1.5 (± 1.6)

*Number of drug classes*					
0	757	413 (54.6%)^c^	344 (45.4%)	76.3 (± 8.0)^b^	2.0 (± 1.9)^b^
1	1166	682 (58.5%)	484 (41.5%)	76.5 (± 7.1)	1.8 (± 1.8)
2	2046	1190 (58.2%)	856 (41.8%)	75.4 (± 6.9)	1.6 (± 1.8)
3	1315	741 (56.3%)	574 (43.7%)	74.0 (± 6.4)	1.4 (± 1.6)
4	312	180 (57.7%)	132 (42.3%)	73.4 (± 5.6)	1.5 (± 1.6)

^a ^The difference between rates of men according to revascularization is statistically significant (p = 0.0003)

^b ^The differences between means (age and Comorbidity index) according to revascularization and number of drug classes are all statistically significant (p < .0001)

^c ^The difference between rates of men according to number of drug classes is not statistically significant (p = 0.3889)

**Figure 1:**
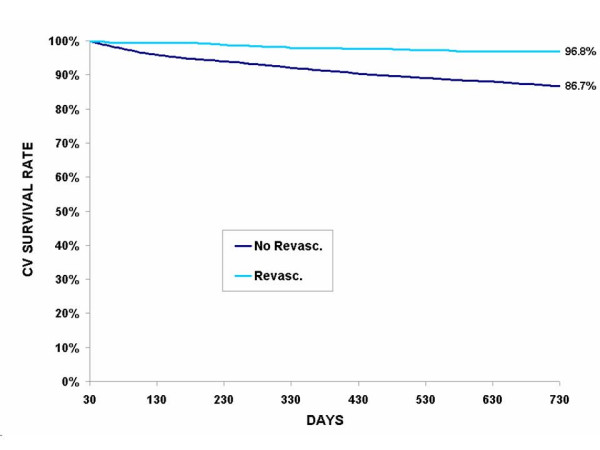
Unadjusted Kaplan-Meier cardiovascular (CV) survival curves according to presence/absence of revascularization during index period among survivors at 30 days (n = 5596)*. * The curves are statistically different (p < .0001).

**Figure 2:**
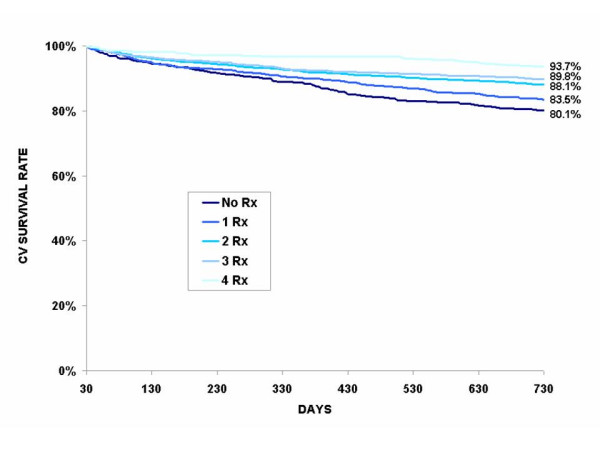
**Unadjusted Kaplan-Meier cardiovascular (CV) survival curves according to the number of cardiovascular drug classes claimed during index period among survivors at 30 days who did not receive a revascularization (n = 4473)**. * The curves are statistically different (p < .0001)

**Figure 3:**
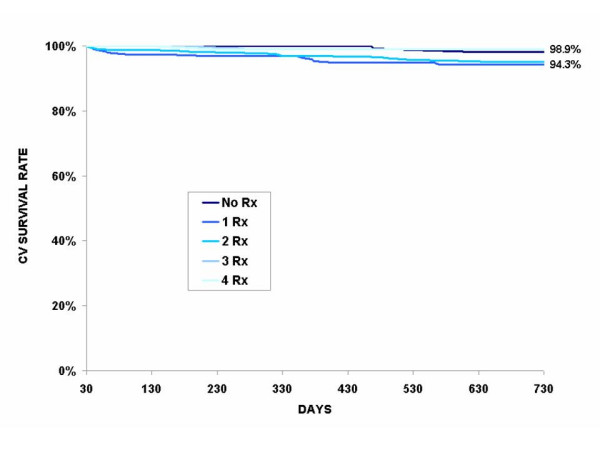
**Unadjusted Kaplan-Meier cardiovascular (CV) survival curves according to the number of cardiovascular drug classes claimed during index period among survivors at 30 days who received a revascularization (n = 1123)**. * The curves are statistically different (p = 0.006)

Of the 5596 cohort patients who survived the index period, 603 (10.8%) died from CV disease and 525 (9.4%) from another cause. Table 3 presents a cross tabulation of the covariates including care (revascularization and cardioprotective drugs) and the 2-year death rates. Here again, younger patients and patients with low comorbidity index have lower CV death rates and non CV death rates at 2 years. More than 1 out of 8 patients (12.7%) on which no revascularization was performed at the index period died from a CV cause after 2 years, and 10.7% from another cause. However the differences in death rates between PTCA and CABG were not statistically significant. Similarly, one out of six (15.5%) patients who did not claim any cardioprotective drugs within the first 30 days after the index hospitalization died from CV disease within 2 years. Here again, patients receiving the most intensive treatment (all four cardioprotective drug classes combined) benefited the most. The same trend is also observed in non CV death rates but to a lesser extent. The cardiovascular survival is clearly demonstrated in Figure 1 to Figure 3 where unadjusted Kaplan-Meier estimates show basically the same relation between the revascularization, the number of cardioprotective drug classes claimed at the index period and the CV survival rate. These curves also demonstrate that CV survival rate is greater for patients with revascularization, regardless of the number of cardioprotective drug classes claimed at the index period. For patients without revascularization, CV survival rate increases with the number of cardioprotective drugs claimed (Figure 2).

**Table 3:** Two-year death rates according to patients' characteristics and exposure to revascularization and medication (n = 5596)

	*Total*	*2-year CV death*	*2-year non-CV death*
*Total*	5596	603 (10.8%)	525 (9.4%)

Mean Age ± SD	5596	79.3 ± 7.3	77.0 ± 7.4
Gender			
Women	2390	291 (12.2%)^a^	215 (9.0%)^d^
Men	3206	312 (9.7%)	310 (9.7%)

*Comorbidity index*			
Mean ± SD (median)	5596	2.6 ± 1.9 (2)	2.6 ± 2.1 (2)
0	2012	83 (4.1%)^b^	89 (4.4%)^b^
1–2	2077	227 (10.9%)	188 (9.0%)
3–4	1111	197 (17.7%)	152 (13.7%)
5–6	318	77 (24.2%)	72 (22.6%)
≥ 7	78	19 (24.4%)	24 (30.8%)

*Revascularization*			
No	4473	567 (12.7%)^c^	478 (10.7%)^c^
Yes	1123	36 (3.2%)	47 (4.2%)
PTCA	767	24 (3.1%)^d^	33 (4.3%)^d^
CABG	356	12 (3.4%)	14 (3.9%)

*Number of drug classes*			
0	757	117 (15.5%)^e^	113 (14.9%)^e^
1	1166	162 (13.9%)	136 (11.7%)
2	2046	207 (10.1%)	178 (8.7%)
3	1315	103 (7.8%)	78 (5.9%)
4	312	14 (4.5%)	20 (6.4%)

^a ^The difference in CV death and in non-CV death rates between genders is statistically significant (p = .0003)

^b ^The difference in CV death and in non-CV death rates between Comorbidity index groups is statistically significant (p < 0.0001)

^c ^The difference in CV death and in non-CV death rates according to presence/absence of revascularization is statistically significant (p < 0.0001)

^d ^The difference in CV death and in non-CV death rates according to PTCA or CABG is not statistically significant (p > 0.05)

^e ^The difference in CV death and in non-CV death rates according to number of drugs classes claimed is statistically significant (p < 0.0001)

Cox proportional hazard models (Table 4) show that adjusted CV death rate increases with age and also with comorbidity index. The hazard ratio (HR) is much lower for patients with revascularization as well as proportionally with the number of classes of cardioprotective drugs claimed.

**Table 4:** Time to death from all cause and time to cardiovascular (CV) death: Cox proportional hazard models (n = 5596)

	***Time to CV death***	
	*Crude HR (95% CI)*	Adjusted HR* (95% CI)

*Age*	1.087 (1.076;1.099)	1.069 (1.057;1.082)
*Male gender*	0.785 (0.669;0.921)	1.010 (0.857;1.190)
*Comorbidity index*	1.315 (1.271;1.362)	1.266 (1.222;1.312)
*Revascularization (PTCA or CABG)*	0.232 (0.165;0.324)	0.368 (0.261;0.518)
*Number of classes of medication*	0.749 (0.696;0.806)	0.853 (0.790;0.920)

* Adjusted for all covariables

**Figure 4:**
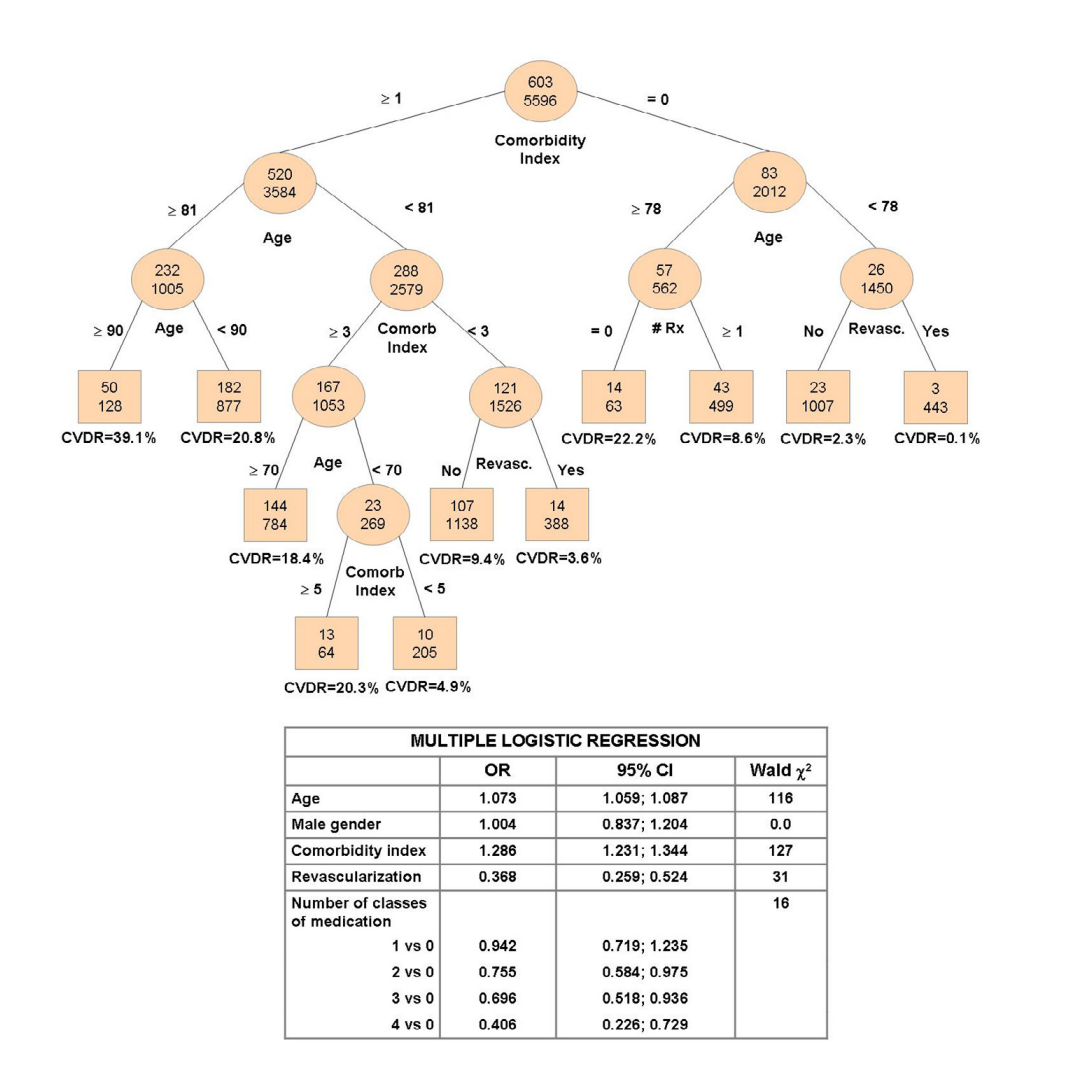
Predictors of 2-year cardiovascular (CV) death: Regression tree* and logistic regression approaches (n = 5596). * The top number in the nodes represents the number of CV deaths and the number below is the total number of individuals in the specific class. CVDR: Cardiovascular death rate.

Regression tree and logistic regression analyses (Figure 4) provide complementary information on variables related to a greater chance of survival 2 years after a MI. The CV death rates vary from 0.1% for younger individuals who had a revascularization with no comorbid conditions to 39.1% for very old patients with comorbidities. For older patients without comorbidities, the use of cardioprotective drugs lowers the 2-year death rates from 22.2% to 8.6%. For younger patients with comorbidities, revascularization decreases the death rates from 9.4% to 3.6%.

## Discussion

The major findings of this population-based cohort study are that CV survival is strongly correlated with revascularization and, at a lesser extent, to the number of classes of cardioprotective drugs claimed. Moreover, the benefit seems to be additive. It is not surprising that populations with and without revascularization differ in age, sex and comorbidity index. However, even after controlling for these factors (Cox proportional hazard model), CV survival remains strongly correlated with revascularization.

Differences between the survival curves may possibly reflect clinical differences unconsidered in the comorbidity index, like non-Q wave myocardial infarction [50,51]. It may also reflect the socio-economical status related to the patient or broader variables like the care setting [52,53], or geographical factors like rural – urban differences [54-56]. This study also demonstrates that survival rate increases with the number of cardioprotective drugs claimed. Similar results were found by Murkherjee et al [40] using an appropriateness algorithm for the use of each secondary pharmacological prevention strategies. In their paper, the score was dependent on the number of medication classes taken among those indicated for the patient. They showed a clear association between an increased score, corresponding to more treatment, and decreased death rates after 6 months.

One can also observe that non cardiovascular survival is increased with revascularization and cardioprotective drugs. However, the extent of this increase is less important than the one observed in cardiovascular survival. We can put forward the hypothesis that the use of revascularization and cardioprotective drugs can reflect a better health care management in general, leading to an increase in non cardiovascular survival as well as in cardiovascular survival.

The major strength of this study is its "naturalistic" population-based cohort. The use of these cohorts can produce results reflecting more closely the real impact of treatment in usual care. Although we acknowledge that validity may possibly be threatened by multiple unrecognized biases, the adjustment for age, sex and comorbidity index, as well as the use of regression tree analysis provide us with more comprehensive knowledge of the relationship between usual care management and survival after a MI.

One of the logistic regression approach disadvantages is that some variables may exert their effect on the whole population while others may be relevant only in specific subgroups (global and local effects). Indeed, for the logistic regression approach, variables enter the equation as main effects. Interactions can be added if they make sense or if based on *a priori *specified hypothesis. This drawback was overcome by using the regression tree approach [47-49] which creates specific subpopulations according to death rates. These regression tree analyses bring forward some considerations on good care management in respect to subpopulations. It also shows that there is not one better treatment for all and that both revascularization and cardioprotective drugs bring significant benefit alone or in combination.

The major limitation of this study is inherent to the use of administrative databases, for example, it was not possible to take in consideration indication and contraindication of revascularization procedures and cardioprotective drugs according to specific clinical condition (STEMI/NSTEMI).

## Conclusion

This study reveals that, when considering usual care for MI, survival is positively correlated with revascularization and the number of cardioprotective drug classes claimed. In this study, important predictors of cardiovascular death after 2 years were the absence of revascularization, older age and higher comorbidity index.

## Competing interests

This project has benefited from an unrestricted grant by Merck Frosst Canada Ltd. This grant was part of a peer reviewed grant obtained from GEOIDE Networks of Centers of Excellence of Canada. None of the authors received salaries, consultation fees or any reimbursement from this company, nor held any shares in this organization.

## Authors' contributions

AV, TN, and JC conceived the study, JC performed the analyses. All authors participated to the writing of the manuscript. All authors read and approved the final version of the manuscript.

## Pre-publication history

The pre-publication history for this paper can be accessed here:


